# Supplementary material for: Evidence supported by Mendelian randomization: impact on inflammatory factors in knee osteoarthritis
Source: Front Med (Lausanne). 2024 May 28;11:1382836. doi: 10.3389/fmed.2024.1382836 (PMC11165061; doi:10.3389/fmed.2024.1382836)
Supplement: Supplementary file 1 [file Data_Sheet_1.ZIP › data/GCST90274845_5e6/845.leaveoneout.pdf]

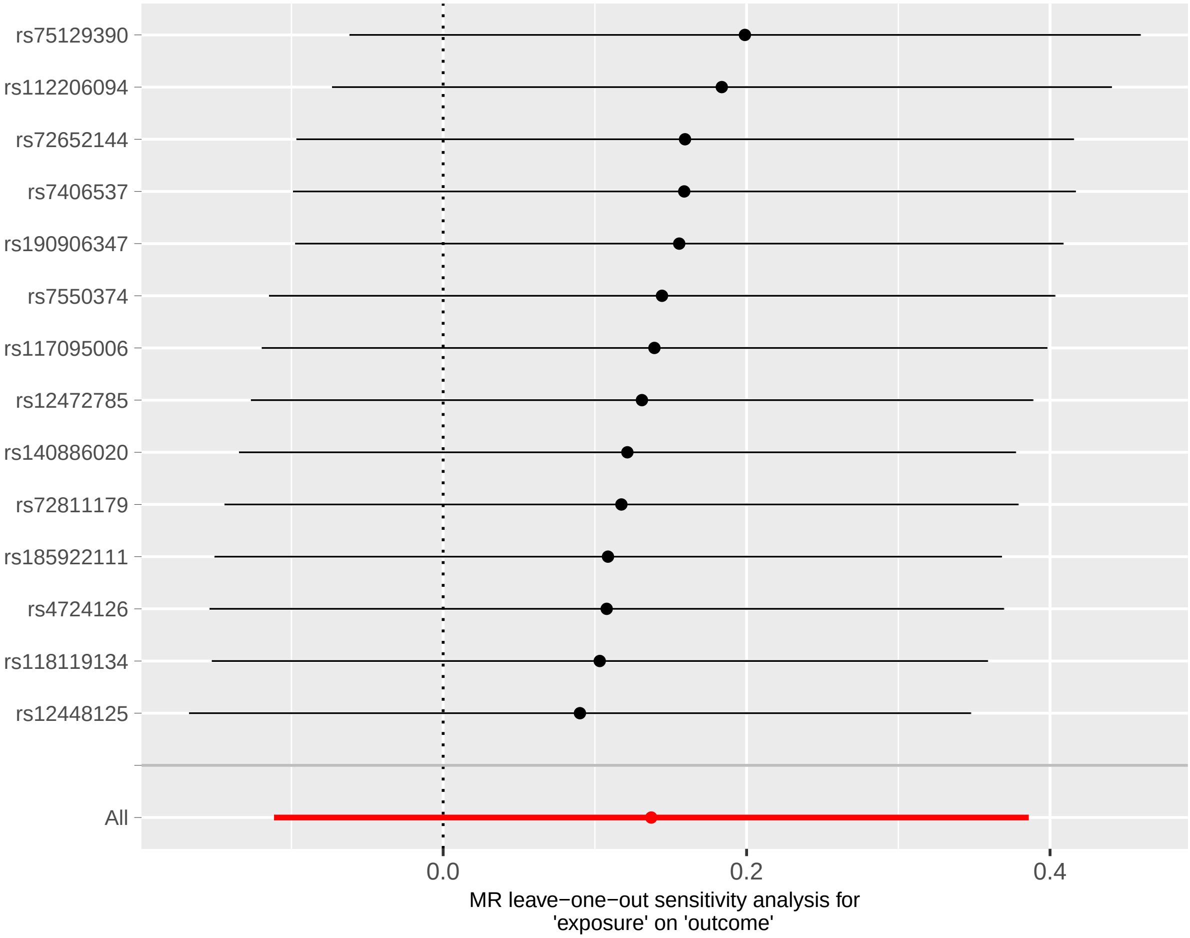
rs75129390
rs112206094
rs72652144
rs7406537
rs190906347
rs7550374
rs117095006
rs12472785
rs140886020
rs72811179
rs185922111
rs4724126
rs118119134
rs12448125
All
0.0
0.2
0.4
MR leave−one−out sensitivity analysis for
'exposure' on 'outcome'
